# A comprehensive method of isolating proteins from serum, cerebrospinal fluid, and hippocampal neurons in rats for proteomic profiling using the LC-MS/MS platform

**DOI:** 10.1016/j.bbrep.2025.102113

**Published:** 2025-06-25

**Authors:** Pratibha Sharma, Rajinder K. Dhamija

**Affiliations:** Department of Neurology, Institute of Human Behaviour and Allied Sciences, New Delhi, India

## Abstract

Neurology research largely utilizes rat brains due to their structural and functional similarities to humans, making them valuable models for studying various neurological conditions. There is growing interest in investigating diseases such as Alzheimer's disease (AD), Parkinson's disease (PD), cognition, and other mental health-related disorders. This has created a need for a comprehensive, combined, and easy-to-follow method to isolate serum, cerebrospinal fluid (CSF), and hippocampal neurons. The hippocampus, responsible for learning and memory, is affected by various neurological and psychiatric disorders. However, obtaining samples like CSF and hippocampal neurons is challenging, especially from small animals like rats. Currently, there is no efficient method for isolating these samples altogether from a single animal, and its use in downstream applications has not been thoroughly tested. We have developed a comprehensive and streamlined method for isolating serum, CSF, and hippocampal neurons from a single animal, suitable for downstream applications such as proteomics and biomarker research. This method involves using high-speed centrifugation instruments and density gradient centrifugation, which are easy to follow. The isolated proteins were identified through mass spectrometry. Our method has been successfully tested for high-throughput applications with small sample volumes, demonstrating its clinical utility. With our simplified approach, proteins in serum, CSF, and neural cells can be studied simultaneously. The method achieves ease of use, cost-effectiveness, and reproducibility, thereby facilitating a better understanding of neurological disorders.

## Highlights

• Cerebrospinal fluid, serum, and brain hippocampal tissue neurons carry important molecules for diagnosis.
• A single method can help ease molecule isolation with better coverage.
• Proteomics can be useful in identifying diagnostic biomarkers and understanding disease mechanisms.
• CSF & hippocampus hold key diagnostic molecules; a unified isolation method can aid biomarker discovery & disease understanding.

## 1 Introduction

Neurodegenerative disorders are a heterogeneous group of disorders posing health-related challenges globally [1]. Alzheimer's disease and Parkinson's disease are the two most common disorders associated with neuronal loss. Animal models are widely used in research to study disease processes, develop disease models, and conduct pre-clinical drug screening [2]. These models provide the insights that can be extrapolated to human health. Rat models such as TgF344-AD, APP NL-G-F knock-in, 6-hydroxydopamine (6-OHDA), and Alpha-synuclein overexpression models are commonly used in the study of neurodegenerative disorders [3,4]. Various sampling methods are employed, including CSF, brain tissue analysis, and blood sampling. The disruption of normal CSF can lead to the development of neurodegenerative disorders, such as AD and PD, ischemic and traumatic brain injury, and neuroinflammatory conditions like multiple sclerosis [5]. CSF has shown potential usefulness in diagnosing AD and PD through the biomarkers Abeta 1–42 and alpha-synuclein, respectively [6,7]. During diseased conditions, neuro-inflammation is mediated by inflammatory cytokines [[8], [9], [10]]. These cytokines are circulated across the blood-brain barrier (BBB) through a saturated transport mechanism, and there is overwhelming evidence supporting the cross-talk between the immune response system and brain pathology [11]. CSF is a clear, colorless body fluid produced in the choroid plexus of the brain's ventricles. It acts as a cushion, providing mechanical and immunological protection to the brain and spinal cord from injury. This makes it an important sample for diagnosing diseases in the central nervous system (CNS).

The brain's hippocampus is crucial for learning and memory. The degeneration of neurons in this region is linked to neurodegenerative and cognitive disorders [12,13]. In AD, the loss of connections and damage to neurons occur in brain areas involved in memory, such as the entorhinal cortex and the hippocampus. The severe shrinkage of the hippocampus, which is responsible for forming new memories, is a significant concern. Neural regeneration therapies have been suggested and studied based on culturing techniques [14,15]. Understanding neurons is essential for comprehending the nervous system and the mechanisms of neurodegeneration.

Rodents, due to their genetic, biological, and behavioral similarities to humans, are commonly used in laboratory work and for sample collections [16]. They are cost-effective and breed quickly, making them ideal for various studies. However, injecting small animals and collecting CSF can be challenging and often results in high mortality rates. Previous methods have presented a poor success rate in producing clear CSF [17,18]. Blood sampling, particularly in studies investigating proteins that cross the BBB, offers a potential non-invasive diagnostic approach [19]. However, the selective permeability of the BBB may complicate the detection of brain-specific markers in the blood. Collecting CSF from rats in the appropriate volume and without blood contamination poses challenges. Nevertheless, this fluid is vital for studying protein interactions and mechanisms involved in various diseases, especially when combined with the isolation of hippocampal neurons.

**Fig. 1:**
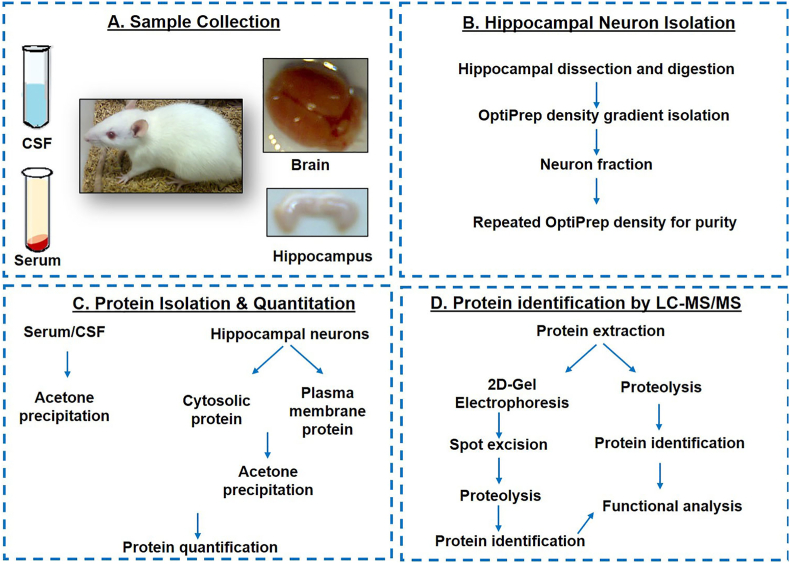
Overview of methodology for isolation and identification of proteins using LC-MS/MS from CSF, serum, and hippocampal neuron cytosolic and plasma membrane.

We aimed to develop a comprehensive sampling protocol tailored for various downstream applications, such as proteomics. This methodology is designed to facilitate protein identification through mass spectrometry by sampling serum, CSF, hippocampal neuron intracellular, and plasma membrane from rats. An overview of the methodology is presented in the flow chart in Fig. 1. By carefully considering the various factors involved, we have ensured that the method is accurate, efficient, and reproducible.

## 2 Materials & methods

### 2.1 A. materials

**Sample:** Thirty Wistar Albino rat strains (weight range of 180–240 g, including 15 male and 15 female rats) were used, as approved by the Institute Ethical Committee of All India Institute of Medical Sciences, New Delhi, India (IAEC No.-647/IAEC/11).

**Reagents:** Micro capillary glass tubes of Length 90 mm with I.D. 1.00 ± 0.10 & O.D. 1.50 ± 0.10 (Mucaps, India), 1.5 mL Eppendorf tubes (Tarsons, India), cotton, general anesthesia-Ketamine and xylazine, Ethanol (Supelco Merck, Germany), pre-sterilized stainless forceps, scissor, and razor (Z225622, Merck, Germany), PMSF Inhibitor Cocktail (G6521, Promega, United States), Trypsin gold, MS grade (Promega, United States), Corning tubes (15 mL, CLS430052, Merck, Germany), BCA Protein Assay kit (Thermo Scientific, USA), Methanol hypergrade for LCMS (1.06035.1000, Merck), Protein ladder (LC5925, Thermofisher Scientific, USA), Peptide Desalting Spin Columns (89852, Pierce, Sweden), Formic Acid LC-MS grade (A11750, Fisher Scientific), Water, LC-MS Grade (85189, ThermoScientific, USA), SYPRO Ruby protein stain (Invitrogen), Peptide desalting spin columns (Pierce, Thermo Scientific, USA), and IPG Buffer, pH 3–10 NL (Amersham Biosciences, USA). 1 mL syringe, 27 Gauge needle, Papain (76216), 9-inch-pasteur-pipette (S6268), OptiPrep density gradient medium (D1556), Sucrose (S8501), Urea (U4883), Thiourea (T8656), CHAPS (850500P), Ammonium bicarbonate (A6141), Dithiothreitol (10197777001), Iodoacetamide (I1149), Acetone (650501), Acetonitrile (34851), Trifluoric acid (T62200), Magnesium choride (208337), Tris Base (252859), Triton-X-100 (X100), HEPES (H3375), Ethylenediaminetetraacetic acid (EDS), Coomassie Brilliant blue G 250 (1.15444), Bromophenol Blue (114391), Glycerol (G5516), Sodium dodecyl sulfate (436143), and Agarose (A6013), were purchased from Sigma Aldrich, America). All other chemicals were of analytical grade.

**Instruments and software:** Gel running buffer (Bio-Rad), pH 3–10 NL IPG strips (Bio-Rad), Multiple Affinity Removal System Spin Cartridge (5188–8825, Agilent), Stereotaxic instrument (LabIndia Instruments), Refrigerated Centrifuge, Swing bucket centrifuge, Ultracentrifuge (Beckman Coulter, Thermo Scientific, USA), SW Ti 41 rotor (Beckman Coulter), Fixed angle rotor (Beckman Coulter), IPGphor 3 and Rehydration tray (Amersham Biosciences, USA), Ruby Gel apparatus (Amersham Biosciences, USA), Nanodrop instrument (ThermoScientific, USA), Mascot Software (Matrix Science), ESI Triple TOF 5600 (SCIEX, USA), and Cytoscape software *version* 3.9.1 (NHGRI, NIH, U.S.).

### 2.2 B. methods

#### 2.2.1 Blood collection and serum isolation

1) The initial experiment was conducted with a Wistar albino rat (180 g). Blood was collected from the retrobulbar plexus or sinus of the rat's eye, performed under general anesthesia [20,21]. The rat was held by the tail, and its head was rotated towards gravity in a circular motion to increase blood flow. The skin around the eye was pulled taut with the thumb and forefinger of the left hand.
2) A fresh and sterile capillary was inserted into the medial canthus of the eye at a 30-degree angle to the nose, and slight pressure was applied to puncture the tissue and access the plexus or sinus.
3) Blood flowed into the capillary and was collected in a clean and sterile 1.5 mL Eppendorf tube. After a few minutes, the same procedure could be repeated to collect blood from the other eye. Approximately 1 ml of blood was collected.
4) The serum samples were left undisturbed at 4 °C and incubated for 1 h to allow the blood to clot. The samples were then centrifuged at 1000 g for 10 min at 4 °C to remove the clot.
5) 120 μL of clear serum was collected, and any blood-contaminated samples were discarded. Aliquots of 100 μL each of serum samples were collected and stored at −80 °C for further use. Serum samples were depleted for high abundant proteins using Multiple Affinity Removal System Spin Cartridges.

#### 2.2.2 CSF collection

1) Intraperitoneal anesthesia was administered to the rat by injecting ketamine (100 mg/kg) and xylazine (10 mg/kg). 1X PBS, pH 7.4, was added up to 1 mL. A toe pinch was not responded to by the rat when properly anesthetized. The appropriateness of the dosage for the rat's weight was ensured, and PBS was added to prevent dehydration.
2) Specially constructed ear bars were placed in the external auditory meatus, and the rat was securely fixed to the Stereotaxic instrument.
3) The rat's head was flexed downward at a 90-degree angle. A depressible surface with the appearance of a rhombus between the occipital protuberances and the spine of the atlas was made palpable. The head hair was cleaned using scissors and a razor. The cleared head was wiped with 70 % Alcohol.
4) An incision was made at the midline of the scalp. The cervico-spinal muscle was reflected, and the atlanto-occipital membrane was exposed.
5) The rat's head was laid down at a 135-degree angle to the body. The atlanto-occipital membrane was punctured by the fire-polished 1 ml syringe with a 27G needle. Non-contaminated CSF was collected in the syringe by gentle aspiration. CSF contaminated with blood was discarded. The amount of CSF collected may vary (80–160 μL) depending on the rat's gender, age, size, and overall health conditions.
6) Thus collected CSF was centrifuged at 14,000 g for 15 min at 4^o^C to remove any cells present in it. It was incubated at 4^o^C for 1 h and stored in 50 μL aliquots at −80^o^C for further use.

#### 2.2.3 Isolation of the hippocampus from the brain

1) After CSF was collected, the rat was immediately euthanized by overdosing on Ketamine and xylazine.
2) The head was decapitated with a guillotine and disinfected with 70 % ethanol. The skin over the top of the skull was dissected to expose it. A scissor was carefully inserted into the spinal canal, and the calvarium on one side was cut nearly to the front. Similarly, the process was repeated on the other side. Care was taken not to damage the brain.
3) With the help of forceps, the base of the skull was grabbed, and the hippocampi were carefully taken. To dissect the hippocampi, the brain was oriented at the dorsal side so that the clear midline of the two hemispheres was visible. The curved forceps were inserted down the dorsal midline to approximately half the depth and were squeezed to sever the cerebral commissure. They were re-inserted with a 2 mm spread into the midline of the midbrain. One hemisphere was gently peeled to the side. The above steps were repeated with the contralateral hippocampus.
4) Both the hippocampi from the rat brain were collected into 2 mL PBS at 4^o^C in a 35 mm diameter petri dish. Three washes with PBS were performed. Care must be taken not to squeeze the tissue as this will affect neuronal isolation.

#### 2.2.4 Isolation of hippocampal neurons

1) After the hippocampus was removed, it was placed on a sterile, pre-wet filter paper with PBS. The hippocampus was sliced into 0.5 mm pieces, and then the tissue slices were transferred into a 15 mL Corning tube containing 5 mL of PBS, which was kept on ice at 4 °C.
2) The 15 mL Corning tube with the tissue was placed in a shaking water bath at 30 °C for 10 min to ensure consistent temperature.
3) 12 mg of papain powder was added to 6 mL of PBS in a 15 mL Corning tube. The tube was kept in a 37 °C shaking incubator for 30 min before use. Any insoluble particles were filtered out, and the resulting solution was sterilized and kept on ice until needed. The solution was freshly prepared for every experiment.
4) The tissue was transferred to a Corning tube containing papain at 30 °C using a wide-bore pipette. It was shaken for 30 min at a rate of 170 rpm, which is equivalent to 0.4 g using the incubator. The tissue was transferred to a 15 mL Corning tube containing as little papain as possible in 2 mL of PBS at 30 °C using a wide-bore pipette. It was allowed to sit at room temperature for 5 min. After 1 min, the supernatant was transferred to a fresh 15 mL Corning tube.
5) The contents were triturated approximately 10 times in 45 s using a 9-inch wide-bore Pasteur pipette. Each trituration step involved drawing the tissue up into a pipette without air bubbles and then immediately emptying the contents back into the tube, again without introducing air bubbles.
6) The sediment from the first tube was resuspended in 2 mL of PBS. The above trituration steps were repeated two more times, combining the supernatants from each trituration.
7) A solution of 60 % iodixanol in water with a density of 1.32 g/mL, known as OptiPrep, was used. Solutions of 7 %, 10 %, 12 %, and 20 % of OptiPrep in PBS were layered in a 15 ml Corning tube. The layers were prepared with the higher-density solution at the bottom and the lower-density solution at the top. The cell suspension was applied to the top of the prepared gradient, allowing the cell suspension to float on top of the gradient.
8) The gradient was centrifuged at 3000 g in a swing bucket centrifuge for 15 min at room temperature.
9) The top 6 mL containing cellular debris was aspirated. Additionally, the top 1 mL of fraction 1, which was enriched with oligodendrocytes, was aspirated.
10) Fraction 3, enriched for neurons, was collected from the lower edge of the dense band to 0.5 mL from the bottom of the tube. Fraction 2, the opaque band, contained cell fragments, neurons, and other cells, while fraction 4 (bottom 0.5 ml and loose pellet) contained microglia.
11) Fraction 3, containing pure neurons, was collected for further applications. To ensure the purity of the neurons, the above-mentioned step was repeated. The fraction of pure neurons was diluted in 5 mL of PBS and centrifuged at 1500 g for 15 min.
12) The obtained pellet was washed three times in PBS and centrifuged at 100 g for 5 min each. The pellet was then resuspended in 100 μL of resuspension buffer (0.25 M sucrose, 10 mM MgCl2, 5 mM Tris-pH 7.4, 1 mM PMSF).
13) Sonication was performed for five cycles with 20s ON and 40s OFF each for 5 min. The suspension was homogenized with ten strokes in 5 min.
14) The sample was centrifuged at 14,000 g at 4^o^C for 15 min. The supernatant was stored at −80 °C for further applications. The supernatant obtained contained cytosol proteins from hippocampal neurons. The pellet was processed further for plasma membrane protein isolation.

#### 2.2.5 Isolation of plasma membrane proteins from hippocampal neurons

1) PBS was used to dilute the pellet, and a sucrose gradient was prepared in ultra-clear tubes. The sucrose gradients were prepared in PBS, pH 7.4, and layered bottom to top with 60 %, 35.5 %, 25.5 %, and 19 %, respectively. Either the bottom or the top of the layer can be used to place the samples, carefully using a 3 mL Pasteur pipette by the side wall of the tube.
2) Ultracentrifugation was performed at 100,000 g for 1.75 h at 4^o^C in a Beckman Coulter SW Ti 41 rotor by overlaying onto sucrose density gradient. Plasma membrane proteins were obtained at the interface of 25.5 % and 35.5 % of sucrose.
3) The plasma membrane fraction was collected and diluted in PBS. The ultracentrifugation step was repeated at 100,000 g at 4^o^C for 1.75 h using a fixed-angle rotor.
4) The supernatant was discarded, the pellet was collected, and it was stored at −80 °C for further experiments.
5. Concentration of CSF, Serum, and hippocampal neuron cytosolic and plasma membrane proteins by the acetone precipitation method
1) Four times the volume of cold (−20 °C) acetone was added to each sample. The tubes were vortexed and incubated for 45 min at −20 °C. The samples were centrifuged at 21,000 g at 4 °C for 10 min.
2) The supernatant was discarded without disturbing the protein pellet. The protein pellet was resuspended in 80 % acetone (pre-chilled) and centrifuged as in step 1.
3) The above steps were repeated twice to obtain the protein pellet. The acetone was completely dried off the protein pellets
4) The protein pellet was dissolved in 1 mL of 10 mM PB, pH 7.4, 0.1 % Triton-X-100. A protease inhibitor cocktail was added to all samples to avoid proteolysis according to sample volume.
5) Rat protein samples were stored at −80 °C in aliquots of 100 μL for each sample source (serum, CSF, etc).

#### 2.2.6 Protein quantification

Protein concentrations in the serum, CSF, plasma membrane proteins of hippocampal neurons, and cytosol proteins of hippocampal neurons were determined by using a BCA protein assay kit.

#### 2.2.7 2D gel electrophoresis

##### 2.2.7.1 Protein sample solubilization

1) Samples of serum, CSF, and hippocampal tissue neuron proteins from cytosol and plasma membranes were collected at 200 μg each. These were further solubilized in lysis buffer (8 M Urea, 3 M Thiourea, 4 % CHAPS). Plasma membrane samples were carefully solubilized in a solubilization buffer.
2) Membrane preparations were resuspended (0.5 mg/mL) in 20 mM HEPES, pH 7.4, 150 mM KCl, 1 mM EDTA, 1 % (w/v) CHAPS, and solubilized at 4 °C for 1 h with end-to-end rotation.
3) The solubilized membrane was centrifuged at 21,000 g for 15 min, and supernatants were collected.

##### 2.2.7.2 Protein sample rehydration

1) A final volume of 250 μL was made with lysis buffer after 1.25 μL of IPG buffer, 1 μL of BPB (from 0.002 % stock), and 0.75 mg of DTT were added to each sample. The tube containing samples was centrifuged at 21,000 g for 2 min and then loaded onto a Rehydration tray.
2) An IPG-strip of pH range 3–10 NL, 13 cm was used for IEF. The samples were carefully covered with the strips for rehydration for 14–16 h.

##### 2.2.7.3 Two-dimensional gel electrophoresis

1) For first-dimension electrophoresis, the IPG strips were rehydrated for isoelectric focusing in an IPGphor 3 system. Total volt-hours of 30,000 VhT were used.
2) For the second dimension, the strips were equilibrated in SDS-equilibration buffer containing 50 mM Tris-HCl, pH 8.8, 6 M urea, 30 % glycerol, 2 % SDS, and 0.02 % bromophenol blue.
3) The strips were first equilibrated for 15 min with 0.05 % DTT prepared in 5 mL of SDS equilibration buffer at RT. The solution was decanted and replaced with 1.25 % iodoacetamide solution, prepared in 5 mL of SDS equilibration buffer, for 15 min at RT.
4) The 10 % polyacrylamide gels were prepared using a Ruby gel apparatus. The strips were carefully loaded on the PAGE and sealed with the help of an agarose solution (0.5 % agarose, 0.002 % BPB).
5) The gel was run at 15 mA for 30 min, followed by 30 mA until the bromophenol blue tracking dye came out of the gel. The gels were first stained using Colloidal Coomassie. The colloidal Coomassie was destained and re-stained using silver stain for compatibility with mass spectrometry.
6) For in-gel proteolysis, the spots of interest were selected, and in-gel proteolysis was used to identify individual proteins. Gel pieces were destained using a 1:1 solution of sodium thiosulfate and potassium metabisulfite, then dehydrated in 50 % acetonitrile and 25 mM ammonium bicarbonate for 20 min (two changes). Further dehydration was done with 100 % acetonitrile for 20 min until the pieces turned white. Acetonitrile was removed, and the gel pieces were lyophilized. The 0.5 μg of trypsin (dissolved in 50 μl of 40 mM ammonium bicarbonate and 10 % acetonitrile) was added to each sample. The samples were kept on ice for 10 min and then incubated at 37 °C for 18 h. 30 μl of 50 % acetonitrile and 0.1 % formic acid were added, incubation was carried out for 20 min, and the supernatant was collected. The extraction was repeated twice, the extracts were pooled, and the samples were lyophilized. These samples may be analyzed by mass spectrometry.

#### 2.2.8 Proteolysis by in-solution trypsinization of proteins

1) The sample pellet was resuspended in 100 μL of 50 mM Ammonium bicarbonate, and the pH was adjusted to 8.0. 5ul of DTT (prepared in 100 mM Ammonium bicarbonate) was added. 100 μg of each serum, CSF, hippocampal intracellular protein, and plasma membrane protein samples for further processing.
2) The samples were boiled for 10 min and kept at RT for 1 h after gentle vortex and spin.
3) 4 μL of Iodoacetamide was added, vortexed, spun, and kept at RT for 1 h.
4) 2 μL (1 μg/μL) of trypsin was added on ice to each sample. The samples were vortexed, spun, and incubated at 37 °C for 16 h. The samples were lyophilized. The samples were lyophilized to dry up completely. Peptide desalting was performed before mass spectrometry analysis.

#### 2.2.9 Peptide desalting, LC-MS/MS analysis, and data analysis

1) Peptide desalting spin columns were purchased and used as per the manufacturer's protocol.
2) The dried peptides were resuspended in 0.1 % formic acid.
3) 2 μL of each sample peptide was used to quantify concentration with a Nanodrop instrument.
4) Label-free quantitation was used, and 2 μg of sample peptide was diluted in 10 μL of 0.1 % formic acid.
5) We equilibrated the column and analytical column with 0.1 % FA for LC parameters. The samples were kept in an autosampler. 1 μg of each digested peptide was injected into the column.
6) The column and analytical column were equilibrated with 0.1 % FA for LC parameters. The samples were kept in an autosampler. 1 μg of each digested peptide was injected into the column.
7) The raw data files of the MS/MS spectrum were obtained and analyzed using Mascot software for protein analysis.
8) Further biological protein interaction networks related to proteins identified from serum, CSF, and hippocampal neurons were analyzed.

## 3 Results and discussions

### 3.1 Isolation of serum and CSF proteins

**Fig. 2:**
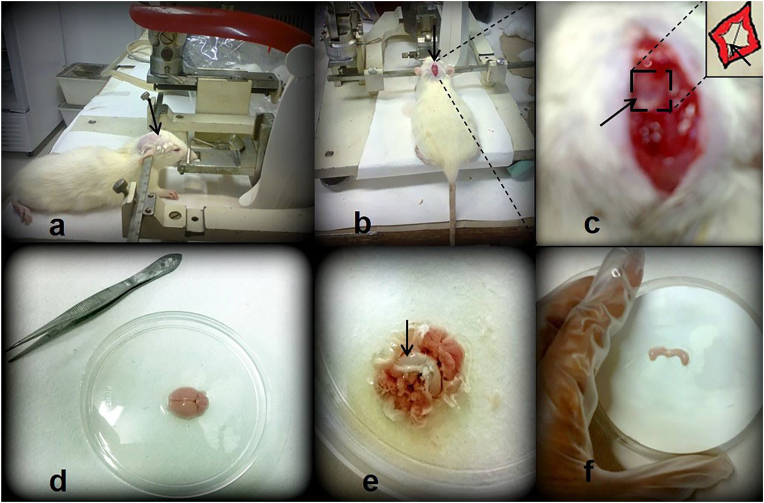
**Methodological steps for the collection of CSF and isolation of the brain hippocampus.** CSF was collected (a,b,c), and hippocampus tissue was isolated (d,e,f). a. Wister albino rat was fixed in the stereotaxic instrument for CSF collection. b. The head hair was cleared, and an incision was made at the scalp's midline. Cervico-spinal muscle was reflected. c. The atlanto-occipital membrane was exposed and punctured for CSF collection. d. The rat brain was isolated. e. The hippocampus tissue was exposed, f. The hippocampus was isolated.

The method for isolating proteins from serum, CSF, and brain hippocampal neurons has been optimized for downstream applications such as proteomics. In our study, thirty Wister albino rat strains (both male and female) were used, and testing was conducted for serum, CSF, and hippocampal neuron isolation (data not shown). Serum is defined as the clear, colorless blood plasma that does not include fibrinogens, white or red blood cells, or clotting factors. Serum and CSF proteins whose expression levels differ in patients with AD (published previously), PD, and dementia compared to controls have been identified in multiple studies [[22], [23], [24], [25]]. The advantage of easy sampling and minimal contamination for biomarker investigations is offered by blood serum. Using this method, 1–2 mL of blood can be collected from a rat per experiment. Sample volume for blood may vary depending on the method used for collection, such as tail vein, jugular vein, and cardiac puncture [21]. CSF can be collected without contamination using general anesthesia. A high success rate in the production of clear CSF has been achieved, which is an improvement over previous methods [17,18]. Following the sampling, the process of protein isolation and analysis using mass spectrometry-based shotgun proteomics has been refined and discussed. Ketamine, a medication providing amnesia, analgesia, and dissociation from the environment, is often combined with xylazine, an α2 adrenergic agonist, to achieve relatively safe, short-term anesthesia [17]. To simplify the collection of CSF from the cisterna magna, rats were properly fixed to a stereotaxic instrument [Fig. 2]. On average, 80–160 μL of CSF with no blood contamination was successfully aspirated from 27 out of 30 tested rats, resulting in a 90 % success rate in CSF sampling. Following serum and CSF collection, rats were promptly sacrificed to isolate the hippocampus and obtain neurons. Plasma membrane proteins were isolated from intracellular, mitochondrial, and microsomal materials using high-speed centrifugation with density gradients. These proteins can be worked on by researchers based on their research interests. Additionally, neuron culture can be performed if necessary [15]. Recently, the comparative isolation method for microglia and astrocytes from mouse brain has been discussed using isolation methods like Magnetic-activated cell sorting (MACS) and Fluorescence-activated cell sorting (FACS) [26]. Their roles in maintaining neuronal circuits and responding to inflammation may be better understood. The detailed method for the isolation of hippocampal tissue neurons and their downstream potential use in biomarker discoveries has been presented.

### 3.2 Isolation of hippocampal neuron proteins

**Fig. 3:**
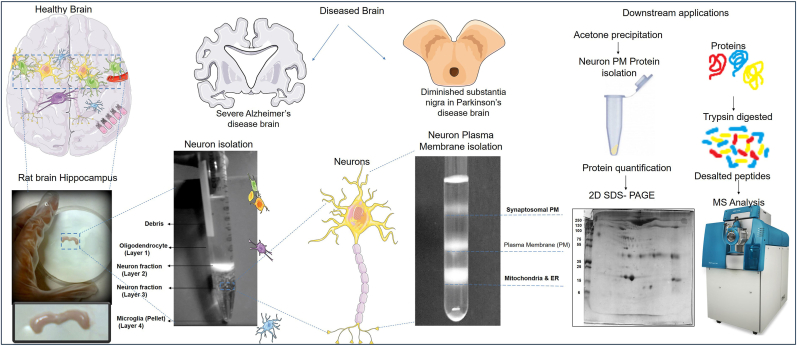
**Isolation of hippocampus and neuron proteins for downstream applications.** Isolation of rat brain hippocampal neurons was performed using Optiprep density gradient. Fraction 3 contained neuron fraction, repeated twice with fraction 3 to obtain the purity of neurons. Isolation of the Hippocampal neuron plasma membrane protein was performed using sucrose density. Hippocampal neuron intracellular and plasma membrane proteins were identified using mass spectrometry, and other downstream applications may be performed. Cartoon images of the brain and neurons were obtained from SMART-Servier Medical ART available at https://smart.servier.com/.

The optical density gradient method was utilized to separate cell debris and microglia from the neuron fraction (Fig. 3). The hippocampal neurons were contained in Fraction 2, but low purity was noted, and contamination by other cells may have occurred. A pure neuron fraction was obtained from one rat hippocampal tissue in Fraction 3 of the gradient [Supplementary data Fig. S1]. The trituration step is considered critical in neuron isolation. The hippocampal slices were treated with papain and triturated carefully and gently. The obtained neuron cells were dissolved in a resuspension buffer, and lysis of the cells was performed using sonication. Sound energy was applied to agitate particles in the sample, disrupt neuron cell membranes, and release cytosolic components through sonication. Dissolution was sped up by breaking intermolecular interactions. The cell suspension was homogenized. This resulted in the extraction of membrane proteins and the solubilization of protein complexes to preserve the protein-protein interactions. Subsequently, the membrane fraction was purified on a discontinuous sucrose gradient between 35 % and 25.5 % sucrose (see Fig. 3). Mitochondrial and ER fractions were removed from the plasma membrane at an interface of 35.5 % and 50 % gradient. Sucrose molecules are large carbohydrate molecules, and their presence may interfere with protein-protein interaction studies. These were removed either by dialysis or by protein precipitation methods. Re-ultracentrifugation was performed after diluting the plasma membrane fractions in PBS at pH 7.4, such that the final concentration of sucrose was less than two percent. 1 mM PMSF was added to the membrane protein, commonly used in protein-solubilization, to deactivate proteases that might digest proteins of interest after cell lysis.

## 4 Downstream application of isolated proteins

### 4.1 2D-SDS PAGE and enzymatic digestion

**Fig. 4:**
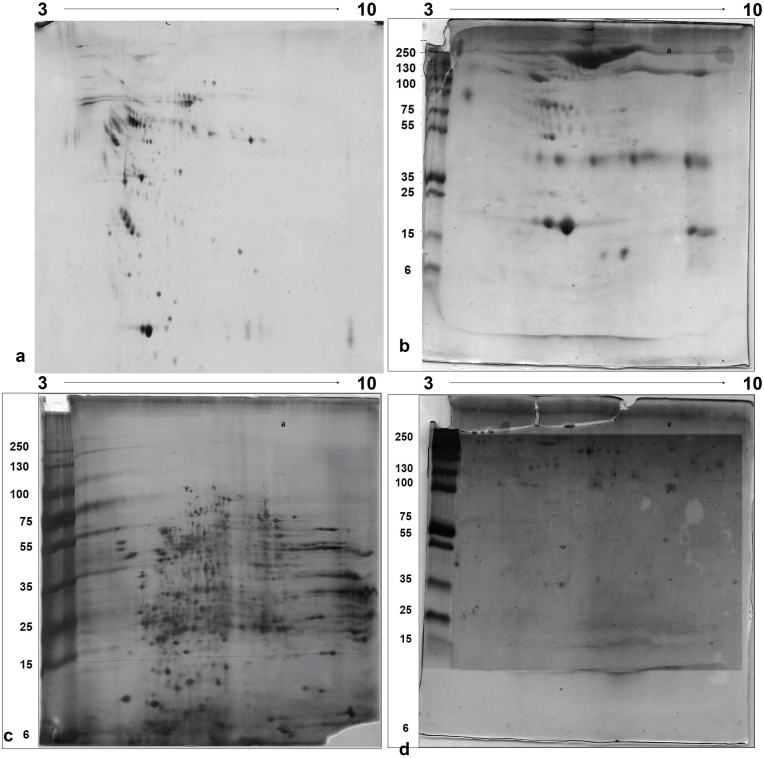
**2D-PAGE of serum, CSF, hippocampal neuron intracellular proteins, and hippocampal neuron plasma membrane proteins was generated.** 10 % 2D-PAGE gel, pH 3–10 NL, 13 cm strip was used. Staining was done with silver stain.

Two-dimensional sodium dodecyl sulfate-polyacrylamide gel electrophoresis (2D SDS-PAGE) is recognized as a reliable and efficient method for separating several hundred to a few thousand proteins. First, separation of the proteins is achieved based on differences in their isoelectric point (pI), and then based on molecular weight (Fig. 4). More control over the outcome of the MS analysis is provided by in-solution digestion, as compared to in-gel digestion, resulting in a greater number of peptides being generated. Identification of low molecular weight proteins from the gels is often difficult. Easier recovery of peptides and adjustments in conditions such as pH, protein concentration, digestion buffer, and proteolytic enzyme are facilitated. However, it may lead to loss of proteins during processing, and samples need to be salt-free before MS analysis. Femtomole quantities of proteins can be identified by these methods. Mass spectrometry was used to identify trypsin-digested peptides.

### 4.2 Protein identification by mass spectrometry

The ESI Triple TOF 5600 (SCIEX) was utilized to detect trypsin-digested peptides from serum, CSF, hippocampal neuron intracellular, and plasma membrane proteins. This instrument is known to be highly effective for analyzing complex proteomics samples. The *Rattus norvegicus* species database was searched using Mascot software. In the serum, CSF, plasma membrane proteins, and cytosol proteins of hippocampal neurons, 115, 92, 147, and 173 proteins were identified, respectively, with a false discovery rate of less than 1 %. A non-redundant list of these identified proteins is provided in Supplementary Table S1–S4. The protein scores from the MS/MS result search are based on the ion scores.

### 4.3 Protein-protein interaction analysis

**Fig. 5:**
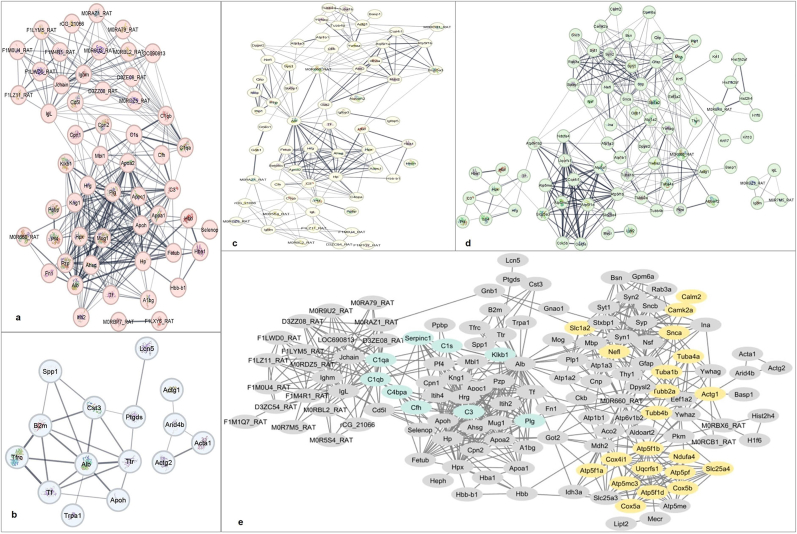
**Protein-protein interactions study of serum, CSF, hippocampal neuron intracellular, and plasma membrane proteins was identified by mass spectrometry.** Serum proteins (a), CSF (b), hippocampal neuron intracellular (c), and hippocampal neuron plasma membrane proteins (d) were included. Protein interaction networks obtained from CSF, serum, hippocampal neuron intracellular, and membrane proteins identified were merged to show protein-protein interactions. The common proteins from various neurodegenerative disease pathways, such as AD and PD, Amyotrophic lateral sclerosis (ALS), Huntington's, and prion disease, are shown in yellow color. The proteins such as C1QA, C1QB, C1S, C3a, C3, C3AR1, C4BPA, CFH, CFHR1, CFHR4, CFHR5, KLKB1, PLG, SERPINC1, TFPI involved in complement and coagulation cascades are shown in cyan color (e). This interaction data was obtained using Cytoscape software vs 3.9.1 and the String plugin.

The protein-protein interaction analysis of proteins identified through mass spectrometry was conducted using Cytoscape v3.9.1 and the String plugin. It was found that many proteins showed similarities in the CSF, serum, and hippocampal intracellular proteins. The protein-protein interaction networks from the four samples were merged, and the individual maps as well as the merged maps are shown in Fig. 5.

### 4.4 Study limitation

The primary strength and limitation are found in the methodology used for the brain hippocampal neurons. Simple-to-follow steps for a combined method to isolate proteins from blood, CSF, and hippocampus neurons have been presented. However, a small sample size and the use of a limited species constrain the study, which may restrict the generalizability of the findings to humans and other animals. High-speed centrifugation instruments and density-gradient centrifugation methods are required by the methodology. Additionally, an older mass spectrometry instrument was utilized, which could limit protein detection sensitivity. A greater number of proteins could be identified by newer, high-tech mass spectrometry instruments. The translational impact of the findings is further restricted due to the need for validation in larger and more diverse sample cohorts.

### 4.5 Future direction and implications in disease mechanism

Transferrin and albumin were found in both CSF and serum, and an association with neurodegenerative disorders might be indicated by their dysfunction [[27], [28], [29], [30]]. Direct contact with the extracellular space of the brain is held by CSF, and biochemical changes that occur inside the brain can be reflected by it [[8], [9], [10]]. 80 % of proteins are commonly shared by CSF and serum. For these reasons, both the CSF and serum are considered for the biomarker discoveries that aid in the early diagnosis of AD. Entry of blood proteins into CSF is allowed by BBB dysfunction. Impaired absorption of proteins and elevations of acute inflammatory mediators, including cytokines reported in neurodegenerative disorders, can result from the CSF flow abnormality. Cytokines are circulated across the BBB by a saturated transport mechanism and are involved in cross-talk with brain pathology [11]. In AD, connections are lost by the neurons, and neuron damage occurs in the parts of the brain involved in memory, including the entorhinal cortex and the hippocampus [12,13]. Severe shrinkage is observed in the hippocampus, an area of the cortex that plays a key role in the formation of new memories. The best approach to study plasma membranes, glial cell-free, and rich fractions from hippocampal neurons of the central nervous system is provided. A combined method for the collection of CSF, serum, and hippocampus neurons has been developed with an improved success rate in producing clear CSF. Approximately 4 h will be taken by the entire process of serum, CSF, and hippocampus neuron isolation. Neural cell plasma membrane proteins have been successfully isolated and identified using mass spectrometry. The method is cost-effective, simple, and reproducible. The study of proteins in serum, CSF, and neural cells is enabled by this method for researching protein cross-talks and neurological disorder mechanisms using specific rat models.

## CRediT authorship contribution statement

**Pratibha Sharma:** Writing – original draft, Visualization, Validation, Software, Methodology, Funding acquisition, Formal analysis, Data curation, Conceptualization. **Rajinder K. Dhamija:** Writing – review & editing, Validation, Supervision, Resources.

## Availability of data and materials

All the data corresponding to our manuscript have been provided in the supplementary data. Any additional data that support the findings of our study will be provided upon reasonable request to the corresponding author.

## Ethical approval and consent to participate

All procedures involving Wistar albino rats were approved by the Institute Ethical Committee of the All India Institute of Medical Sciences, New Delhi (IAEC No. 647/IAEC/11). All experiments were conducted in accordance with the ARRIVE guidelines (Animal Research: Reporting of In Vivo Experiments) and adhered to the Guidance on the operation of the Animals (Scientific Procedures) Act 1986, as well as the EU Directive 2010/63 for the protection of animals used for scientific purposes. These procedures also align with the NIH (National Research Council) Guide for the Care and Use of Laboratory Animals or those established by an equivalent internationally recognized body.

## Consent for publication

Not applicable.

## Funding

This project work was supported by a fellowship grant from the 10.13039/501100001411Indian Council of Medical Research, India (Code-13340), and a grant from the Department of Health Research, India (R.12013/16/2024) to P.S.

## Declaration of competing interest

On behalf of all Co-Authors, I shall bear full responsibility for the submission. I confirm that all authors listed on the title page have contributed significantly to the work, have read the manuscript, attest to the validity and legitimacy of the data and its interpretation, and agree to its submission.

Also, this work has not been submitted to any other journal for publication.

## Data Availability

Data will be made available on request.
