# Supplementary material for: A comprehensive method of isolating proteins from serum, cerebrospinal fluid, and hippocampal neurons in rats for proteomic profiling using the LC-MS/MS platform
Source: Biochem Biophys Rep. 2025 Jun 25;43:102113. doi: 10.1016/j.bbrep.2025.102113 (PMC12264605; doi:10.1016/j.bbrep.2025.102113)
Supplement: Multimedia component 1 [file mmc1.pdf]

Supplementary Data

Table S1: Proteins identified by mass spectrometry from hippocampal neuron intracellular proteins sample

Score	% Cov (95)	Uniprot id	Protein Name	Peptides (95%)
69.65	42.0	P12346	Serotransferrin	35
50.42	14.2	Q6MG79	Complement component 4	28
31.08	33.4	Q5M7V3	LOC367586 protein	19
38.24	16.6	G3V9R2	Protein Cfh	18
34.99	35.7	P20059	Hemopexin	18
39.01	17.1	Q91YB6	Complement inhibitory factor H	18
27.03	29.9	Q5M842	IgG-2a protein	17
22.07	7.0	M0RBJ7	Complement C3	14
23.97	12.0	P06687	Sodium/potassium-transporting ATPase subunit alpha-3	11
15.80	16.1	Q5I0J0	Immunoglobulin heavy chain	11
19.45	24.3	P85108	Tubulin beta-2A chain	10
17.82	62.7	Q05175	Brain acid soluble protein 1	9
14.22	24.4	Q4KM66	LOC500183 protein	9
15.49	20.7	G3V7C6	RCG45400	8
14.86	38.0	I7FKL4	Myelin basic protein transcript variant 1	8
12.01	44.3	P01836	Ig kappa chain C region	8
10.70	55.8	P20767	Ig lambda-2 chain C region	7
14.00	19.5	P63259	Actin, cytoplasmic 2	7
10.96	12.7	F1LP05	ATP synthase subunit alpha	6
10.00	11.2	I1T7F1	GLT1a splice variant	5
9.54	15.2	M9MMN0	Protein Ighg3	5
10.00	34.0	P02091	Hemoglobin subunit beta-1	5
9.67	10.1	Q569B3	Igh-6 protein	5
7.17	7.2	Q5M891	C4b-binding protein alpha chain	5
7.94	33.1	B1H216	Hemoglobin alpha	4
8.01	14.1	M0R660	Protein RGD1565368	4
6.07	31.4	P04638	Apolipoprotein A-II	4
8.00	9.5	P09951	Synapsin-1	4
6.95	25.2	P11517	Hemoglobin subunit beta-2	4
6.85	9.1	Q99PS8	Histidine-rich glycoprotein	4
3.99	9.5	P04636	Malate dehydrogenase	3
6.60	37.1	P06765	Platelet factor 4	3
6.21	7.6	P13233	2',3'-cyclic-nucleotide 3'-phosphodiesterase	3
5.11	22.7	P19939	Apolipoprotein C-I	3
7.11	5.1	P47942	Dihydropyrimidinase-related protein 2	3
6.16	11.6	P60203	Myelin proteolipid protein	3
4.13	6.2	Q5M7T5	Protein Serpinc1	3
5.77	6.7	Q6P9V9	Tubulin alpha-1B chain	3
4.00	11.4	F1M229	Uncharacterized protein	2
2.85	4.5	G3V6D3	ATP synthase subunit beta	2

4.29	15.9	M0R4L7	Histone H2B	2
2.00	6.8	M0RBP7	Uncharacterized protein	2
2.76	6.6	P06866	Haptoglobin	2
4.25	6.8	P15473	Insulin-like growth factor-binding protein 3	2
4.66	8.8	P24090	Alpha-2-HS-glycoprotein	2
3.15	5.4	P59215	Guanine nucleotide-binding protein subunit alpha	2
3.17	4.4	Q5M8A0	Kng2 protein	2
1.72	4.6	Q6IRS6	Fetub protein	2
1.34	10.8	Q99ME0	CXC chemokine RTCK1	2
2.05	2.7	B0BMT0	RCG47746, isoform CRA_a	1
2.00	9.4	D3ZC54	Uncharacterized protein	1
0.40	3.3	D3ZCA0	Proline synthetase co-transcribed	1
0.79	0.5	D4A8Q2	Protein Kndc1	1
2.00	10.3	F1LYU4	Uncharacterized protein	1
2.00	12.1	F1LZ11	Uncharacterized protein	1
2.00	13.5	F1M0U4	RCG21092	1
0.76	6.4	F1M1Q7	Uncharacterized protein	1
0.68	4.7	G3V7N9	Complement C1q subcomponent subunit B	1
2.17	0.7	G3V7W2	Fibronectin	1
2.03	8.5	G3V8Z5	Uncharacterized protein	1
2.05	4.8	M0R7B4	Protein LOC684828	1
0.67	2.7	M0RAS8	Elongation factor 1-alpha	1
2.30	1.7	M0RCB1	Uncharacterized protein	1
2.63	13.7	M0RDZ5	Uncharacterized protein	1
1.00	15.0	O09019	Nestin	1
0.90	2.1	P00507	Aspartate aminotransferase	1
0.85	1.0	P02770	Serum albumin	1
1.66	2.9	P07335	Creatine kinase B-type	1
1.41	4.6	P07340	Sodium/potassium-transporting ATPase subunit beta-1	1
1.06	5.9	P10888	Cytochrome c oxidase subunit 4 isoform 1	1
2.01	1.5	P19527	Neurofilament light polypeptide	1
1.51	3.7	P22057	Prostaglandin-H2 D-isomerase	1
1.12	3.0	P24594	Insulin-like growth factor-binding protein 5	1
2.00	3.5	P54311	Guanine nucleotide-binding protein G(I)/G(S)/G(T) subunit beta-1	1
2.00	1.7	P61765	Syntaxin-binding protein 1	1
0.82	3.2	P61983	14-3-3 protein gamma	1
2.23	5.7	P63102	14-3-3 protein zeta/delta	1
2.00	3.0	Q6AY07	Fructose-bisphosphate aldolase	1
2.00	3.4	Q6IRH6	Slc25a3 protein	1
2.00	4.9	Q6MFX9	Myelin oligodendrocyte glycoprotein	1
2.00	2.2	Q6P7S6	Clusterin	1
2.00	2.9	Q6PAH0	Apolipoprotein E	1
2.00	1.0	Q7TMA9	Aa1249	1
2.30	2.0	Q91WX0	Complement factor H-related protein	1
2.00	5.5	Q91Y34	Platelet phospholipase A2	1
2.00	0.8	Q920H8	Hephaestin	1

2.00	3.0	Q99NA5	Isocitrate dehydrogenase [NAD] subunit alpha	1
1.89	1.2	Q9ER34	Aconitate hydratase	1
0.59	5.8	Q9Z1I0	Shc transforming protein	1

Table S2: Proteins identified by mass spectrometry from hippocampal neuron plasma membrane proteins sample

Score	% Cov (95)	Uniprot id	Protein Name	Peptides (95%)
32.76	20.8	P06687	Sodium/potassium-transporting ATPase subunit alpha-3	16
28.02	27.2	P12346	Serotransferrin	12
22.06	29.9	G3V7C6	RCG45400	12
22.06	15.3	P06686	Sodium/potassium-transporting ATPase subunit alpha-2	10
17.44	8.9	Q7TP24	Ba1-667	9
17.30	44.1	I7FKL4	Myelin basic protein transcript variant 1	10
15.04	21.3	P63259	Actin, cytoplasmic 2	10
14.78	19.3	V9GZ85	Protein LOC100361457	10
14.28	23.6	P85108	Tubulin beta-2A chain	11
14.13	17.3	Q99PS8	Histidine-rich glycoprotein	7
12.00	14.7	P31596	Excitatory amino acid transporter 2	7
11.45	8.2	Q6IMF3	Keratin, type II cytoskeletal 1	6
11.23	12.1	P11275	Calcium/calmodulin-dependent protein kinase type II subunit alpha	6
10.20	14.4	Q6P9V9	Tubulin alpha-1B chain	5
10.00	47.3	Q05175	Brain acid soluble protein 1	5
9.70	13.0	Q5M7V3	LOC367586 protein	5
9.38	8.7	Q6IFW6	Keratin, type I cytoskeletal 10	5
8.70	14.8	P13233	2',3'-cyclic-nucleotide 3'-phosphodiesterase	4
8.12	9.6	Q8K5B5	Glutamate transporter GLT1b	6
8.00	9.0	I1T7F1	GLT1a splice variant	4
8.00	11.0	Q5I0J0	Immunoglobulin heavy chain (Gamma polypeptide)	4
7.53	10.2	P20059	Hemopexin	4
6.39	17.2	D4ABT0	Guanine nucleotide-binding protein G(o) subunit alpha	3
6.09	6.7	Q5XIF6	Tubulin alpha-4A chain	3
6.06	13.8	G3V6D3	ATP synthase subunit beta	3
6.05	22.3	P62804	Histone H4	3
6.00	5.6	P61765	Syntaxin-binding protein 1	3
6.00	17.1	Q4KM66	LOC500183 protein	3
5.52	7.0	P09951	Synapsin-1	3
5.31	3.0	Q6P6Q2	Keratin, type II cytoskeletal	3
5.12	19.7	P02091	Hemoglobin subunit beta-1	3
5.03	34.0	P01836	Ig kappa chain C region, A allele	3
4.92	13.5	P47819	Glial fibrillary acidic protein	2

4.52	5.6	B0BMT0	RCG47746, isoform CRA_a	2
4.44	15.9	M0R4L7	Histone H2B	3
4.28	22.9	P06765	Platelet factor 4	2
4.25	19.8	M0R660	Protein RGD1565368	2
4.13	11.6	P60203	Myelin proteolipid protein	3
4.11	28.2	Q71DI1	Dermcidin	3
4.07	4.6	D3ZQQ5	Dynamin-1	2
4.05	15.8	P07340	Sodium/potassium-transporting ATPase subunit beta-1	2
4.00	13.5	P54311	Guanine nucleotide-binding protein G(I)/G(S)/G(T) subunit beta-1	3
4.00	13.7	G3V7Y3	ATP synthase subunit delta	2
4.00	21.1	M0RDZ5	Uncharacterized protein	2
4.00	8.3	P60881	Synaptosomal-associated protein 25	2
4.00	22.6	Q63754	Beta-synuclein	2
4.00	9.4	Q6P9Y4	ADP/ATP translocase 1	2
4.00	5.0	Q812E9	Neuronal membrane glycoprotein M6-a	2
3.74	9.9	P01830	Thy-1 membrane glycoprotein	2
3.22	15.4	Q6I8Q6	Histone H2A	2
3.11	17.1	P63102	14-3-3 protein zeta/delta	2
2.80	4.0	M0RBJ7	Complement C3	1
2.52	6.5	G3V8Q2	Alpha-internexin	1
2.41	17.8	Q9WUW2	Vesicle associated membrane protein 2B	1
2.36	14.8	R9PY00	Vesicle-associated membrane protein 2	1
2.27	8.1	P00762	Anionic trypsin-1	6
2.27	1.0	Q5EBC0	Inter alpha-trypsin inhibitor, heavy chain 4	1
2.22	34.3	P37377	Alpha-synuclein	1
2.20	1.6	Q6IFU8	Keratin, type I cytoskeletal 17	1
2.11	17.2	P10888	Cytochrome c oxidase subunit 4 isoform 1	1
2.04	6.1	P19527	Neurofilament light polypeptide	1
2.04	4.2	P47942	Dihydropyrimidinase-related protein 2	1
2.04	8.1	Q6IRH6	Slc25a3 protein	1
2.00	12.4	P12075	Cytochrome c oxidase subunit 5B	2
2.00	22.0	B2RZD6	Ndufa4 protein	1
2.00	0.4	D3ZZ51	Protein Prrc2b	1
2.00	10.2	M0R7G5	Protein LOC100911905	1
2.00	2.7	M0RAS8	Elongation factor 1-alpha	1
2.00	15.0	O09019	Nestin	1
2.00	5.3	P06349	Histone H1t	1
2.00	4.7	P20788	Cytochrome b-c1 complex subunit Rieske	1
2.00	16.9	P29419	ATP synthase subunit e	1
2.00	2.4	P62632	Elongation factor 1-alpha 2	1
2.00	4.9	Q5RK07	Igh-6 protein	1
2.00	4.7	Q6AY07	Fructose-bisphosphate aldolase	1
2.00	3.1	S5RZM8	Cytochrome c oxidase subunit 2	1
1.89	3.8	P11980	Pyruvate kinase isozymes M1/M2	1
1.70	1.4	P62815	V-type proton ATPase subunit B	1

1.66	3.6	P63012	Ras-related protein Rab-3A	1
1.64	3.3	P07825	Synaptophysin	1
1.55	3.8	F1M882	Secretory carrier-associated membrane protein 5	1
1.46	1.0	F1MA98	Nucleoprotein TPR	1
1.42	1.6	F1LPY7	Trans-2-enoyl-CoA reductase, mitochondrial	1
1.22	6.3	B1H216	Hemoglobin alpha, adult chain 2	1
1.16	0.0	Q9QUL6	Vesicle-fusing ATPase	1
1.07	8.9	P11240	Cytochrome c oxidase subunit 5A	1
0.77	5.6	Q0QF43	Malate dehydrogenase	1
0.75	2.6	D4A4M0	Protein Shisa6	1
0.71	0.7	D3ZJF8	Protein Fcgbp	1
0.54	0.5	P21571	ATP synthase-coupling factor 6, mitochondrial	1
0.51	0.5	P19527	Neurofilament light polypeptide	1
0.50	0.5	P61983	14-3-3 protein gamma	1
0.44	0.5	P21707	Synaptotagmin-1	1
0.36	0.5	D3ZKH2	Protein Mon2	1
0.31	0.5	D3Z9Z2	Putative lipoyltransferase 2, mitochondrial	1
0.29	0.5	Q9Z1I0	Shc transforming protein	1
0.23	0.5	P20767	Ig lambda-2 chain C region	1
0.21	0.5	F1LXD6	Protein Pitpnm2	1
0.20	0.5	Q66HG1	Cyclin-D-binding Myb-like transcription factor 1	1
0.10	0.5	G3V733	Synapsin-2	1
0.09	0.5	D4ABV5	Calmodulin	1
0.08	0.5	D3ZV39	Protein Armcx4	1
0.08	0.5	M0RBX6	Histone H3	1
0.08	0.5	R9PXT6	Focal adhesion kinase 1	1
0.07	0.5	D4AA54	Protein Plekhh1	1
0.06	0.5	Q6PAH0	Apolipoprotein E	1
0.05	0.5	D3ZSV6	DEAH (Asp-Glu-Ala-His) box polypeptide 33 (Predicted)	1
0.05	0.5	D4A3W6	Dual specificity phosphatase 16 (Predicted)	1
0.05	0.5	D4A9W1	Protein Ccdc88c	1
0.05	0.5	G3V984	Protein bassoon	1
0.05	0.5	Q71S46	ATP synthase F(0) complex subunit C3, mitochondrial	1

Table S3: Proteins identified by mass spectrometry from serum sample

Total Score	% Cov (95)	Uniprot id	Protein Name	Peptides (95%)
118.49	36.7	M0RBJ7	Complement C3	63
61.48	40.4	P12346	Serotransferrin	33
48.80	50.7	P20059	Hemopexin	30
60.02	14.8	P04937	Fibronectin	29
36.44	49.5	Q5M7V3	LOC367586 protein	29
33.23	41.9	Q5M842	IgG-2a protein	23

39.86	17.7	Q71SA3	Thrombospondin 1	19
18.69	24.8	Q4VBH1	Ighg protein	18
18.69	26.5	Q569B4	Ighg protein	18
29.35	25.8	P08934	Kininogen-1	16
27.82	24.8	Q569B3	Igh-6 protein	16
29.89	27.6	P02770	Serum albumin	15
16.89	27.8	Q4KM66	LOC500183 protein	14
25.62	31.2	Q5M8A0	Kng2 protein	14
25.14	37.5	P06866	Haptoglobin	13
10.75	63.2	P01836	Ig kappa chain C region	11
20.90	5.5	Q6MG79	Complement component 4	9
10.98	56.3	B1H216	Hemoglobin alpha	7
12.24	5.4	G3V9R2	Protein Cfh	7
12.20	42.2	P02091	Hemoglobin subunit beta-1	7
12.01	14.1	Q99PS8	Histidine-rich glycoprotein	7
9.41	33.3	P11517	Hemoglobin subunit beta-2	6
9.65	53.9	P20767	Ig lambda-2 chain C region	6
8.97	42.1	P19939	Apolipoprotein C-I	5
9.75	3.9	Q63041	Alpha-1-macroglobulin	5
6.14	5.1	D3ZFH5	Protein Itih2	4
7.21	41.2	F1M0U4	RCG21092	4
8.18	11.9	Q5U3X5	Fgl2 protein	4
7.13	10.1	Q6P7S6	Clusterin	4
6.73	12.8	Q6PAH0	Apolipoprotein E	4
5.14	26.6	F1LZ11	Uncharacterized protein	3
2.29	23.1	F8SQR6	Immunglobulin heavy chain variable region	3
6.00	33.3	P04638	Apolipoprotein A-II	3
5.85	37.1	P06765	Platelet factor 4	3
4.73	11.7	P24090	Alpha-2-HS-glycoprotein	3
6.55	9.0	Q4KM75	CD5 antigen-like	3
4.87	11.6	Q64599	Hemiferrin	3
4.70	18.9	Q6PDV1	Lysozyme 2	3
4.17	5.5	Q9EPH1	Alpha-1B-glycoprotein	3
4.63	7.0	Q9EQV8	Carboxypeptidase N catalytic chain	3
4.48	17.1	E9PSU8	Uncharacterized protein	2
3.12	0.6	F1LMV6	Protein Dsp	2
2.86	17.7	F1M1R0	Uncharacterized protein	2
2.88	11.4	F1M229	Uncharacterized protein	2
2.35	14.6	F1M3Y4	Protein RGD1564184	2
3.11	14.0	G3V8Z5	Uncharacterized protein	2
2.23	19.3	M0R628	Uncharacterized protein	2
1.75	0.7	M0R6Z9	Protein Myo18b	2
4.17	7.7	P04639	Apolipoprotein A-I	2
4.57	8.8	P19999	Mannose-binding protein A	2
3.83	4.7	P25236	Selenoprotein P	2
1.80	1.3	Q03626	Murinoglobulin-1	2
3.22	3.3	Q5FVS2	Kallikrein B	2
3.21	4.3	Q6QI47	LRRGT00161	2

4.00	2.0	Q7TMA9	Aa1249	2
1.61	1.2	Q91YB6	Complement inhibitory factor H	2
1.73	19.8	Q99ME0	CXC chemokine RTCK1	2
2.11	2.7	B0BMT0	RCG47746, isoform CRA_a	1
0.72	4.0	D3Z8A1	REVERSED Protein Tctex1d1	1
0.48	5.3	D3ZE08	Uncharacterized protein	1
2.00	11.1	D3ZEP5	Protein Igkv19-93	1
0.68	0.6	D3ZH42	REVERSED Protein Mov10l1	1
0.90	6.5	D3ZPL2	Uncharacterized protein	1
0.57	1.2	D3ZWU1	Bromodomain containing 3	1
0.52	0.9	D4AC99	CTF18	1
2.00	2.9	F1LPR6	Uncharacterized protein	1
3.36	2.3	F1LQT4	Protein Cpn2	1
2.00	13.0	F1LWD0	Uncharacterized protein	1
2.04	18.5	F1LYM5	Uncharacterized protein	1
2.00	10.3	F1LYU4	Uncharacterized protein	1
1.42	9.6	F1M4R1	Uncharacterized protein	1
2.11	7.7	F1M5L5	Uncharacterized protein	1
1.72	10.4	F1M663	Uncharacterized protein	1
2.00	2.8	F1M6N0	Protein LOC686143	1
2.57	9.4	G3V6G1	Immunoglobulin joining chain	1
1.62	1.7	G3V7L3	Complement C1s subcomponent	1
2.00	4.7	G3V7N9	Complement C1q subcomponent subunit B	1
2.56	0.9	G3V9J1	Uncharacterized protein	1
0.62	2.4	M0R660	Protein RGD1565368	1
2.00	5.7	P31720	Complement C1q subcomponent subunit A	1
2.00	8.5	Q32PY8	Protein Sbsn	1
2.00	2.5	Q4G030	C1r protein	1
1.46	1.4	Q4V8G6	Methyltransferase-like 3	1
0.97	2.9	Q5I0M1	Apolipoprotein H	1
0.67	11.8	Q5RK13	Igf1 protein	1
1.44	2.3	Q6IRS6	Fetub protein	1
1.80	1.2	Q7TP84	Ab1-346	1
1.65	0.5	Q7TPK2	Ac2-120	1
0.61	5.8	Q9Z1I0	Shc transforming protein	1

Table S4: Proteins identified by mass spectrometry from CSF sample

Total Score	% Cov (95)	Uniprot id	Protein Name	Peptides (95%)
656.12	10.0	D3ZNR4	Sushi, nidogen and EGF-like domain-containing protein1	36
650.45	28.0	P04639	Apolipoprotein A1	16
425.24	26.8	P04592	Amyloid beta A4	18
387.56	26.7	Q7TMA5	Apolipoprotein B 100	17
313.67	20.4	P01026	Complement C3	8
293.00	20.2	P05371	Clusterin	9
252.00	20.1	Q99PS8	Histidine rich glycoprotein	7

86.00	20.0	P027770	Serum albumin	7
69.06	19.8	Q63514	C4b-binding protein alpha chain	7
17.03	22.1	P02767	Transthyretin	12
16.06	22.3	P05371	Apoliporotein J	17
16.06	22.3	P08649	Complement C4	17
15.62	13.9	G3V8D4	Apolipoprotein CII	8
11.64	17.2	Q7Z5F4	Protease serine 4 isoform B	12
11.64	17.9	Q8N2U3	PRSS3 protein	12
11.01	8.8	P06759	Apolipoprotein CIII	6
10.89	52.1	P01946	Haptoglobin chain	5
10.00	20.2	P02651	Apolipoprotein AIV	6
9.00	9.5	P12346	Serotransferrin	1
9.00	9.5	Q99376	Transferrin receptor protein 1	1
6.04	19.2	P14841	Cystatin-C	1
6.02	52.1	P20059	Hemopexin	5
6.02	12.5	G3V7Q8	Cationic trypsinogen	1
4.00	10.2	Q6GMX3	IGL protein	2
3.85	10.3	P22057	Prostaglandin-H2 D-isomerase	6
2.59	6.4	A2NUT2	Lambda-chain (AA -20 to 215)	1
2.59	6.5	C6KXN3	Lambda light chain of human immunoglobulin surface antigen-related protein	1
2.59	6.4	P08721	Osteopontin	1
2.59	6.4	Q6GMW3	IGL protein	1
2.59	6.4	Q6GMX4	IGL protein	1
2.59	6.4	Q6IPQ0	IGL protein	1
2.59	6.4	Q6PIQ7	IGL protein	1
2.59	6.4	Q6PJG0	Uncharacterized protein	1
2.59	6.4	Q8N355	IGL protein	1
2.59	6.4	Q8N5F4	IGL protein	1
2.31	8.4	P07151	Beta-2-microglobulin	1
2.00	6.4	A0A5E4	Uncharacterized protein	1
2.00	14.3	A0M8Q9	C1 segment protein	1
2.00	4.1	A6XMV6	Secreted phosphoprotein 1	1
2.00	4.0	B7Z351	cDNA FLJ54682, highly similar to Osteopontin	1
2.00	7.0	B9A064	Immunoglobulin lambda-like polypeptide 5	1
2.00	4.8	C4B6Q2	Osteopontin	1
2.00	4.5	F2YQ21	Osteopontin-D	1
2.00	16.0	P01834	Ig kappa chain C region	1
2.00	8.8	P06911	Epididymal-specific lipocalin-5	1
2.00	14.1	P0CF74	Ig lambda-6 chain C region	1
2.00	14.1	P0CG04	Ig lambda-1 chain C regions	1
2.00	14.1	P0CG05	Ig lambda-2 chain C regions	1
2.00	14.1	P0CG06	Ig lambda-3 chain C regions	1
2.00	4.1	P10451	Osteopontin	1
2.00	4.3	P10451-2	Isoform B of Osteopontin	1
2.00	4.5	P10451-3	Isoform C of Osteopontin	1

2.00	4.5	P10451-4	Isoform D of Osteopontin	1
2.00	4.3	P10451-5	Isoform 5 of Osteopontin	1
2.00	4.2	P35572	Insulin-like growth factor-binding protein 6	1
2.00	7.8	Q0KKI6	Immunoblobulin light chain	1
2.00	4.8	Q3LGB0	Osteopontin	1
2.00	6.4	Q567P1	IGL protein	1
2.00	6.4	Q5CZ94	Putative uncharacterized protein DKFZp781M0386	1
2.00	7.3	Q5EFE6	Anti-RhD monoclonal T125 kappa light chain	1
2.00	6.5	Q5FWF9	IGL protein	1
2.00	6.3	Q6DHW4	Uncharacterized protein	1
2.00	6.4	Q6GMV7	Uncharacterized protein	1
2.00	6.4	Q6GMV8	Uncharacterized protein	1
2.00	6.4	Q6GMW4	IGL protein	1
2.00	6.4	Q6GMW6	Uncharacterized protein	1
2.00	7.2	Q6GMX0	Uncharacterized protein	1
2.00	6.4	Q6IN99	IGL protein	1
2.00	6.4	Q6NS95	IGL protein	1
2.00	6.4	Q6P2J1	Uncharacterized protein	1
2.00	6.4	Q6P5S3	Uncharacterized protein	1
2.00	7.2	Q6P5S8	IGK protein	1
2.00	6.4	Q6PIK1	IGL protein	1
2.00	7.2	Q6PIL8	IGK protein	1
2.00	7.2	Q6PJF2	IGK protein	1
2.00	10.8	Q6PJR7	IGL protein	1
2.00	6.4	Q7Z2U7	Uncharacterized protein	1
2.00	6.4	Q8NEJ1	Uncharacterized protein	1
2.00	7.1	Q8TCD0	Uncharacterized protein	1
2.00	14.1	Q8TCJ5	Putative uncharacterized protein DKFZp667J0810	1
2.00	6.4	Q96E61	Uncharacterized protein	1
1.82	2.6	P26644	Beta-2-glycoprotein 1	1
1.43	5.1	P63259	Actin, cytoplasmic 2	1
1.43	2.7	P63269	Actin, gamma-enteric smooth muscle	1
1.43	3.9	P68136	Actin, alpha skeletal muscle	1
1.43	0.9	Q6RI86	Transient receptor potential cation channel subfamily A member 1	1
1.13	38.1	A0N4V7	HCG2039797	1
0.15	0.0	B4DZK1	cDNA FLJ50036, weakly similar to Myosin-11	1
0.15	0.9	F5H149	Coiled-coil domain-containing protein 180	1
0.15	1.2	Q6QD51	Coiled-coil domain-containing protein 80	1
0.15	0.9	Q9JKB5	AT-rich interactive domain-containing protein 4B	1
0.15	1.2	Q9P1Z9-4	Isoform 4 of Coiled-coil domain-containing protein 180	1

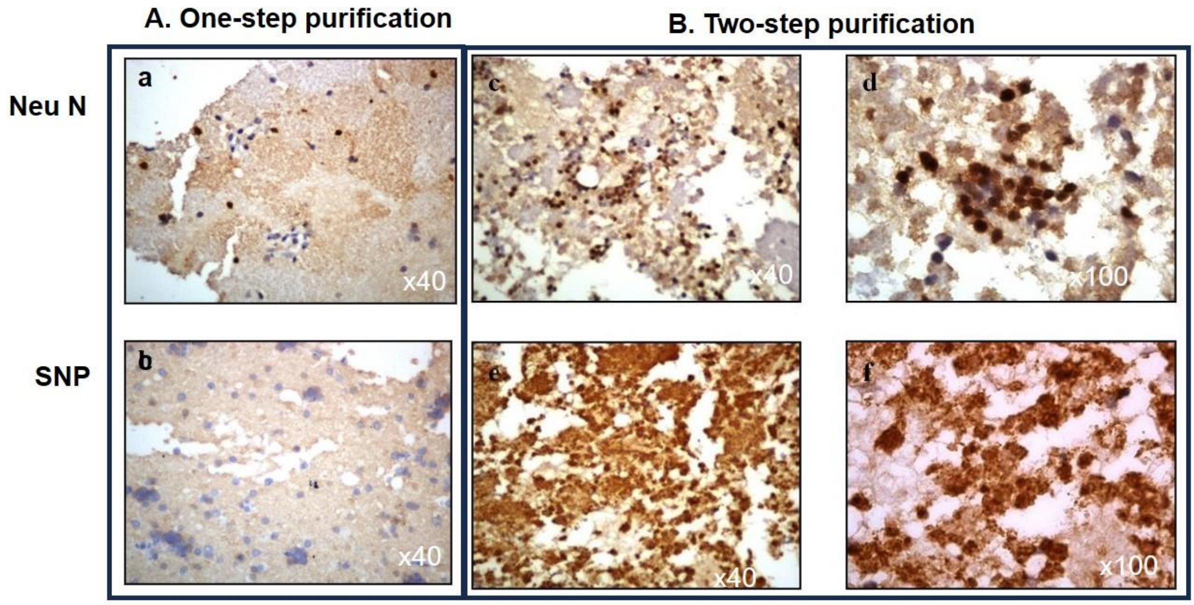

Figure S1: The comparison of neurons obtained in Fraction 3 after one step of Optiprep density gradient A (a, b) and two steps of repeated Optiprep density gradient of Fraction 3 B (c, d, e), are represented. Figure A(a) represents IHC-stained immunoreactive cells for NeuN, a marker for identifying neurons. A(b) represents the immunoreactivity to SNP, a marker for synaptic vesicles in neurons. B (c &d) represents IHC-stained NeuN marker, and B (e & f) represents the neurons obtained after using additional steps for purity by a two-step process of repeated Optiprep density gradient at magnification x40 and x100.

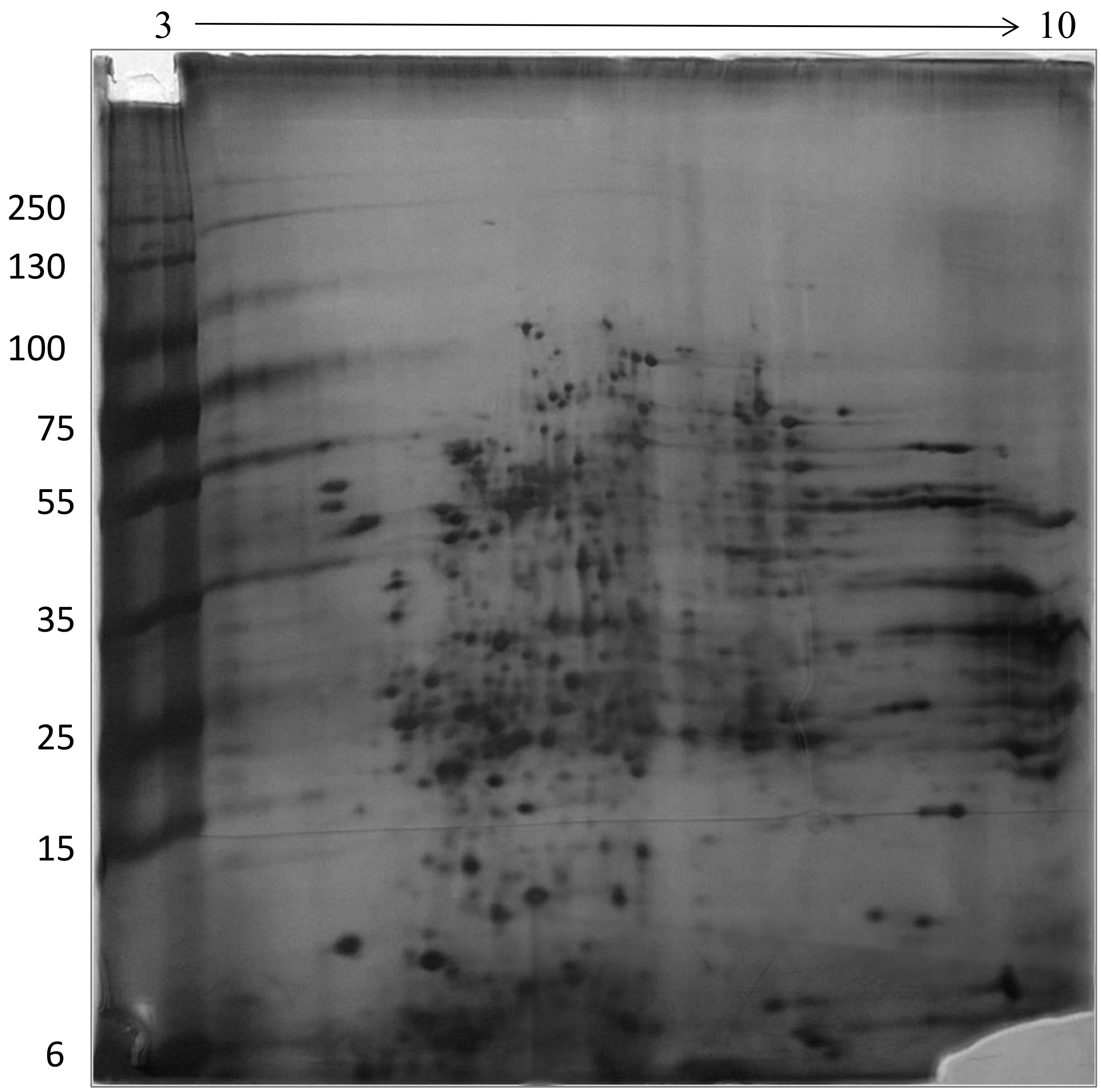

Figure S2: 2D PAGE of Hippocampal neuron intracellular proteins (HNIC). HNIC 2D PAGE, 10% gel, pH 3-10 NL, 13cm strip.

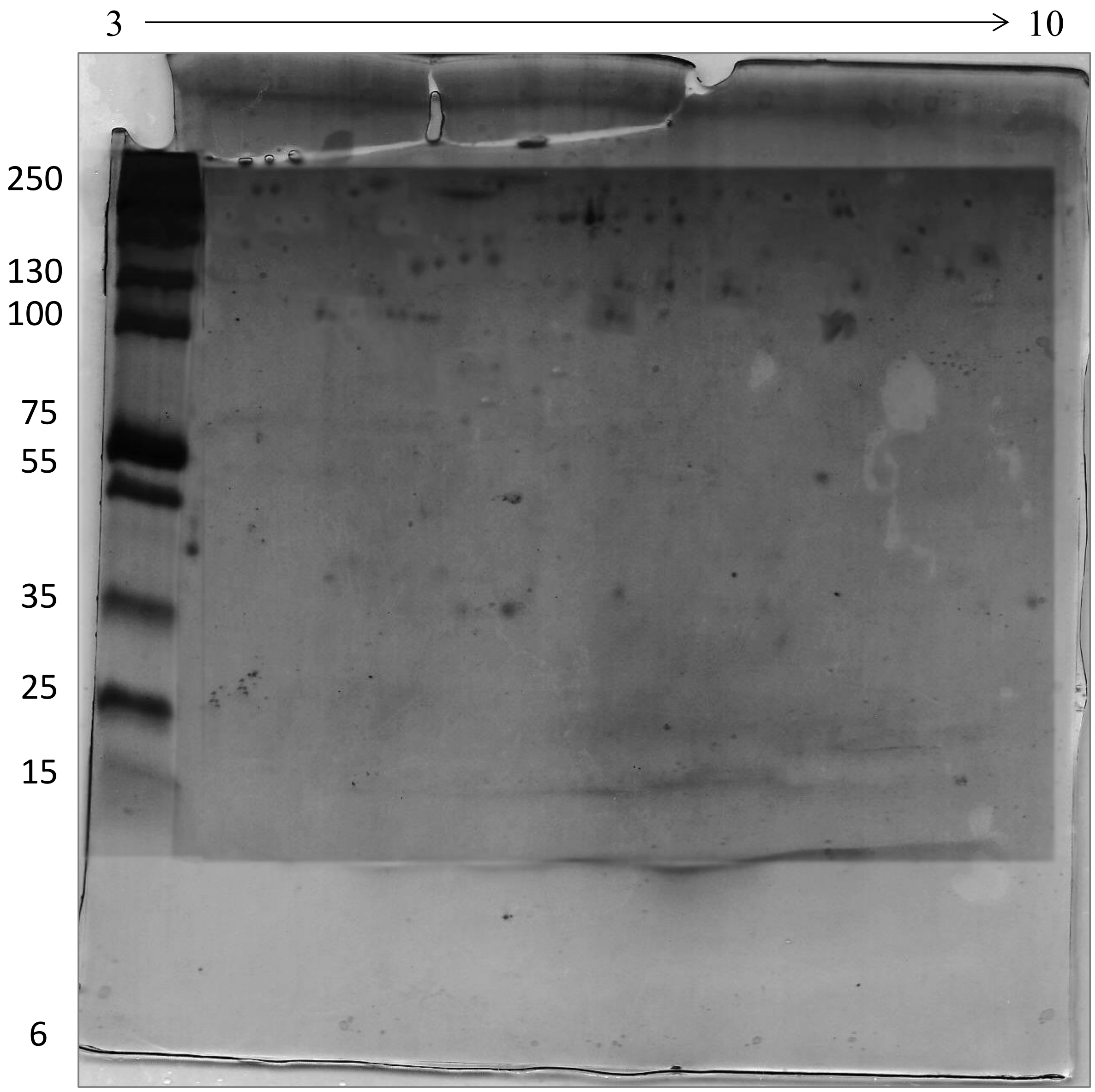

Figure S3: 2D PAGE of hippocampal neuron plasma membrane protein (HNPM). HNPM 2D PAGE, 10% gel, pH 3-10 NL, 13cm strip.

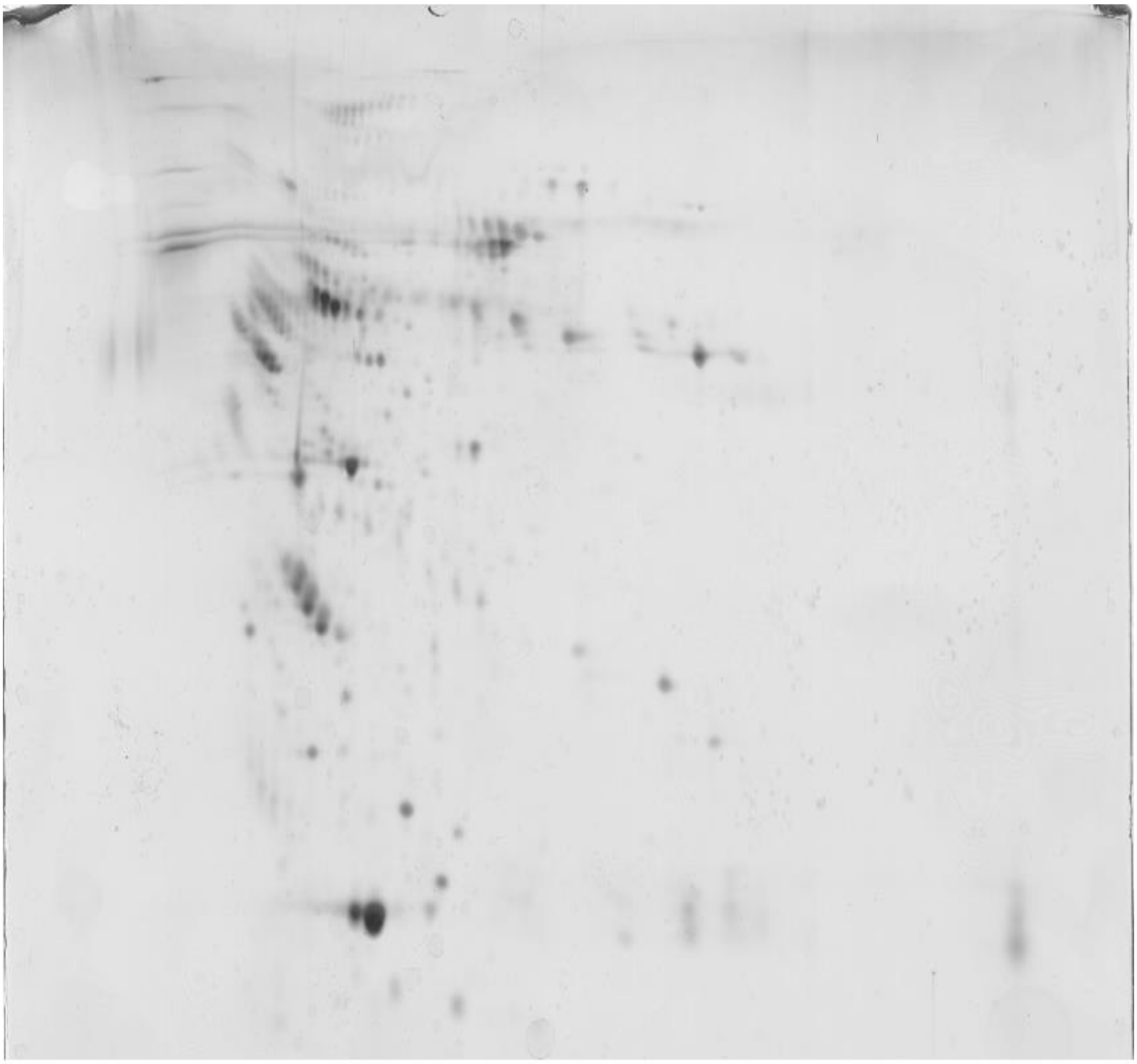

Figure S4: 2D PAGE of depleted serum protein 2D PAGE, 10% gel, pH 3-10 NL, 13cm strip.

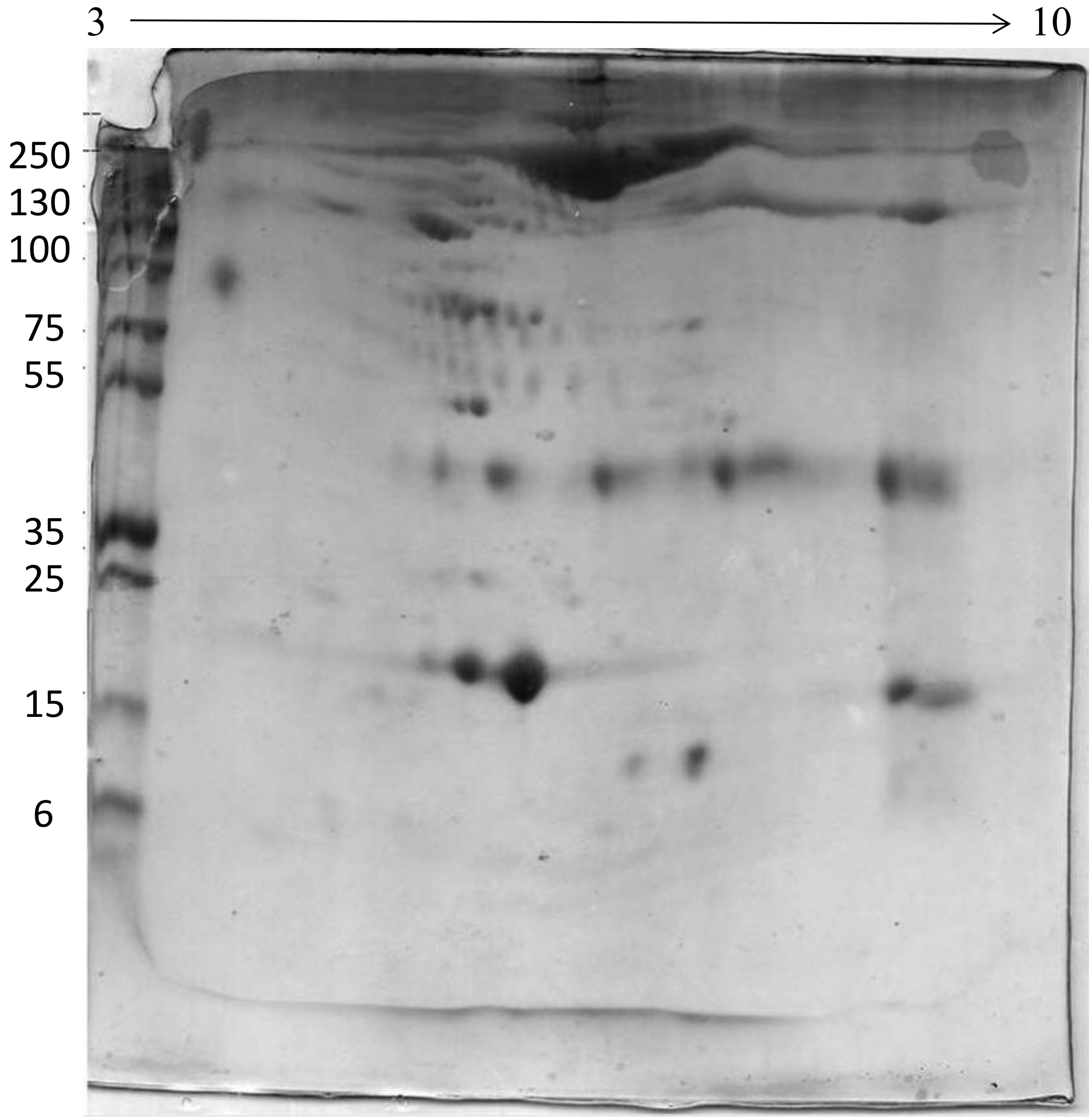

Figure S5: 2D PAGE of cerebrospinal fluid (CSF) protein normal. 10% 2D-PAGE gel of CSF, pH 3-10 NL, 13cm strip.
